# Systematic review of methods used in meta-analyses where a primary outcome is an adverse or unintended event

**DOI:** 10.1186/1471-2288-12-64

**Published:** 2012-05-03

**Authors:** Fiona C Warren, Keith R Abrams, Su Golder, Alex J Sutton

**Affiliations:** 1 Peninsula College of Medicine and Dentistry, St Luke’s Campus, University of Exeter, Exeter, EX1 2LU, UK; 2 Department of Health Sciences, Adrian Building, University of Leicester, University Road, Leicester, LE1 7RH, UK; 3 Centre for Reviews and Dissemination (CRD), University of York, York, YO10 5DD, UK

## Abstract

**Background:**

Adverse consequences of medical interventions are a source of concern, but clinical trials may lack power to detect elevated rates of such events, while observational studies have inherent limitations. Meta-analysis allows the combination of individual studies, which can increase power and provide stronger evidence relating to adverse events. However, meta-analysis of adverse events has associated methodological challenges. The aim of this study was to systematically identify and review the methodology used in meta-analyses where a primary outcome is an adverse or unintended event, following a therapeutic intervention.

**Methods:**

Using a collection of reviews identified previously, 166 references including a meta-analysis were selected for review. At least one of the primary outcomes in each review was an adverse or unintended event. The nature of the intervention, source of funding, number of individual meta-analyses performed, number of primary studies included in the review, and use of meta-analytic methods were all recorded. Specific areas of interest relating to the methods used included the choice of outcome metric, methods of dealing with sparse events, heterogeneity, publication bias and use of individual patient data.

**Results:**

The 166 included reviews were published between 1994 and 2006. Interventions included drugs and surgery among other interventions. Many of the references being reviewed included multiple meta-analyses with 44.6% (74/166) including more than ten. Randomised trials only were included in 42.2% of meta-analyses (70/166), observational studies only in 33.7% (56/166) and a mix of observational studies and trials in 15.7% (26/166). Sparse data, in the form of zero events in one or both arms where the outcome was a count of events, was found in 64 reviews of two-arm studies, of which 41 (64.1%) had zero events in both arms.

**Conclusions:**

Meta-analyses of adverse events data are common and useful in terms of increasing the power to detect an association with an intervention, especially when the events are infrequent. However, with regard to existing meta-analyses, a wide variety of different methods have been employed, often with no evident rationale for using a particular approach. More specifically, the approach to dealing with zero events varies, and guidelines on this issue would be desirable.

## Background

There is increasing concern regarding adverse outcomes of therapeutic interventions, which may be addressed by bringing together evidence from multiple sources [1]. The Cochrane Collaboration, in its remit to undertake systematic reviews, has considered adverse events as an area worthy of consideration as a defined clinical area that requires specific methodology [2], but the emphasis was placed on the non-statistical elements, such as choice of outcomes, study types and search strategies. The use of quantitative evidence synthesis methods with adverse events specifically in mind has been considered some time ago [3], but there is little empirical information collated on meta-analytic approaches within adverse event meta-analyses.

A recent review of systematic reviews of adverse events in response to drug interventions included 43 review articles published in 2006 [4]. However, the statistical methods could only be reviewed in the 24 of these that included a meta-analysis. This review also considered the search strategies used in the systematic reviews of adverse events, and this field has been better documented since it is the focus of several further studies [5-9].

The aim of this current systematic review is to investigate the meta-analytic methodology used where the primary outcome of the meta-analysis was an adverse or unintended event, with the aims of identifying which methods have been used and the challenges faced by authors. A wide range of therapeutic interventions are considered, including drugs, surgical procedures, devices, vaccinations, dietary interventions, anaesthetics and public health programmes. It is hoped that this review will assist in development and standardisation of methods in future and we discuss the areas of priority for further research to facilitate the development of guidelines to support meta-analysis of adverse and unintended outcomes data.

## Methods

The dataset of meta-analyses for this current survey was identified using a database of systematic reviews collected previously [7]. The aim of this previous study was to develop and evaluate search strategies for the retrieval of systematic reviews, which may or may not include a meta-analysis, where the primary outcome was an adverse event (or unintended but positive event) resulting from a clinical intervention. The databases searched (both electronically and by hand) in the previous study were the Database of Abstracts of Reviews of Effects (DARE) and the Cochrane Database of Systematic Reviews (CDSR). Details of the search strategy have been described [7].

These search strategies yielded a total of 257 systematic reviews (246 from DARE plus 11 Cochrane reviews), published between 1994 and 2006. The initial searches were updated, yielding a further 20 systematic reviews on adverse events [9], producing 277 in total.

From the systematic reviews above, references were selected for inclusion in the current review according to the following criteria: 

1. some form of quantitative synthesis (or test for heterogeneity with intention to perform a quantitative synthesis if appropriate) must be performed using more than one observed estimate of effect;

2. the study group of interest must have received some form of clinical intervention with *intended* or *potential* therapeutic effect; and

3. the full study report must be available in English.

A quantitative data synthesis may take the form of a pooled estimate, a confidence interval, quoting a *p*-value only, or performing a meta-regression. Studies entailing only qualitative evidence synthesis, although having an essential role to play in the assessment of adverse events, were not included in this review, which is exclusively aimed at statistical synthesis methods. Meta-analyses of unintended or adverse reactions associated with non-interventional activities, for example recreational drug use, were excluded.

**Table 1:** Number of included reviews by year of publication

**Year of publication**	**No. included reviews (%)**^**1**^	**Published in journal**	**Cochrane review**	**Other publication type**
1994	4 (2.4)	4	0	0
1995	8 (4.8)	8	0	0
1996	9 (5.4)	9	0	0
1997	15 (9.0)	15	0	0
1998	16 (9.6)	16	0	0
1999	19 (11.4)	18	1	0
2000	11 (6.6)	11	0	0
2001	22 (13.3)	22	0	0
2002	21 (12.7)	17	2	2
2003	22 (13.3)	20	2	0
2004	13 (7.8)	10	3	0
2005	4 (2.4)	0	4	0
2006	2 (1.2)	0	2	0

^1^Out of 166 included reviews.

Included reviews were evaluated on multiple aspects. These included type of intervention, number of primary studies incorporated, number of individual meta-analyses performed and source of funding (when determining sponsorship, any form of commercial funding was counted as such, even if other sources of funding were also present; similarly, government sponsorship took precedence over academic). The statistical aspects included general meta-analysis methods and how specific challenges were addressed. These included (i) dealing with sparse data (a count of zero events in one arm of study or zero events across the whole study); (ii) statistical heterogeneity; (iii) dissemination biases (primarily publication bias); (iv) variable quality of primary studies; and (v) use of individual patient data (IPD).

## Results

### Description of included reviews

In total, 166 reviews including a meta-analysis fulfilled all criteria and were included in this systematic review [10-175]. Of these, 14 were Cochrane reviews, the others were published in a wide variety of medical journals or were reviews published by health agencies. Table 1 shows a breakdown of number of included reviews by publication year and type.

The interventions being evaluated for adverse events were similarly diverse; by far the largest category was drug interventions (69.9%, 116/166). The next largest category was surgical interventions (8.4%, 14/166). Other types of intervention, such as forms of anaesthesia, devices, and diagnostic procedures, each accounted for fewer than 5% of the included reviews. Multiple interventions (often multiple therapies for cancer being assessed simultaneously) were considered by 5.4% (9/166) of included reviews.

**Table 2:** Maximum and minimum numbers of contributing estimates for meta-analyses in the same included review

**Maximum no. data observations^**1**^**	**Minimum no. data observations**	**No. included reviews (%)^**2**^**
2−5	2−5	17 (10.4)
6−10	2−5	34 (20.7)
6−10	6−10	6 (3.7)
11−20	2−5	50 (30.5)
11−20	6−10	5 (3.0)
11−20	11−20	4 (2.4)
>20	2−5	29 (17.7)
>20	6−10	12 (7.3)
>20	11−20	3 (1.8)
>20	>20	4 (2.4)

^1^A single primary study may contribute more than one data observation, hence number of data observations may not be the same as number of primary studies.

^2^Out of 164 included reviews with at least one pooled estimate and number of data observations stated.

With regard to sponsorship, the largest number of included reviews were academically sponsored (45.8%; 76/166). Commercial sponsorship accounted for 16.3% (27/166), while 30.1% (50/166) were sponsored by some form of government body. Other sponsorship sources provided funding for 7.2% (12/166), while funding source was not stated for one included review.

Of the 166 included reviews, all except one included some overall combined estimate. The one included review that did not report a combined estimate included a meta-regression for dose–response with no overall pooled estimate of effect size.

The number of meta-analyses performed in each included review was also very variable. In many cases a large number of meta-analyses were performed, with 44.6% (74/166) of studies having more than 10 meta-analyses. By comparison, 19.3% (32/166) of studies had 6−10 meta-analyses, 29.5% (49/166) had 2–5 meta-analyses, 6.0% (10/166) had only one meta-analysis while 0.60% (1/166) had none (meta-regression only).

The number of primary cohorts (contributing a data observation to the meta-analysis) is shown in Table 2. This table indicates that many included reviews performed multiple meta-analyses with varying numbers of data observations derived from primary studies, possibly reflecting multiple outcomes or subgroup analyses.

**Table 3:** Percentages of included reviews by year including assessment of heterogeneity, quality and dissemination bias

**Year**	**Total no. included reviews**	**Heterogeneity (no. (%))**	**Quality (no. (%))**	**Dissemination bias (no. (%))**
1994	4	2 (50)	1 (25)	1 (25)
1995	8	7 (88)	1 (13)	4 (50)
1996	9	8 (89)	1 (11)	6 (67)
1997	15	12 (80)	8 (53)	5 (33)
1998	16	14 (88)	3 (19)	10 (63)
1999	19	18 (95)	8 (42)	12 (63)
2000	11	9 (82)	4 (36)	4 (36)
2001	22	19 (86)	8 (36)	15 (68)
2002	21	16 (76)	13 (62)	8 (38)
2003	22	16 (73)	10 (45)	15 (68)
2004	13	11 (85)	7 (54)	5 (38)
2005	4	4 (100)	4 (100)	3 (75)
2006	2	2 (100)	2 (100)	1 (50)

### Statistical methodology aspects

Table 3 sets out the number of included reviews that cover heterogeneity, quality and dissemination bias, by year of publication, to examine any changes over time.

#### Outcome measures

Table 4 shows the proportions of included reviews using different effect measures. This is important because the choice of outcome measure may in itself influence the meta-analysis method and results.

In Table 4 the option ‘More than one’ was only selected in the eventuality that there was no obvious primary outcome metric, but instead at least two outcome metrics that appeared to receive approximately equal importance in the meta-analyses. Otherwise, the primary outcome measure was recorded even if there were other outcome metrics used in secondary analyses such as sensitivity analyses.

Many of the outcomes being reported in the primary studies were binary, thus lending themselves to analysis by odds ratio or relative risk, as seen in Table 4. A difference scale (for example risk difference), as opposed to a ratio scale, was chosen only infrequently. Only 9.6% of studies presented more than one outcome measure. This indicated that either more than one clinical outcome was being investigated, requiring a different outcome metric, or that the authors had presented analyses for the same clinical outcome on equal terms for different outcome metrics, possibly intended as a comparison between the two.

**Table 4:** Measure of effect

**Measure of effect**	**No. included reviews**	**Percent included reviews^**1**^**
Comparative measures (between interventions)		
Odds ratio	55	33.1
Relative risk	51	30.7
Risk difference	8	4.8
Mean difference	6	3.6
Standardised mean difference	7	4.2
Percent difference	2	1.2
Non-comparative measures		
Correlation	2	1.2
Probability (or percent)	13	7.8
Mean difference	2	1.2
Percent difference	3	1.8
Multiple measures		
More than one	16	9.6
Other measures		
Other	1	0.6

^1^Out of 166 included reviews.

#### Meta-analysis methodology

Another major area of interest was the methodology used for the meta-analysis itself. Table 5 sets out the meta-analysis method used to combine studies along with numbers and percentages.

**Table 5:** Meta-analysis methods

**Meta-analysis method**	**No. included reviews**	**Percent included reviews^**1**^**
Standard fixed effect^2^	54	32.5
Other fixed effect^2^	9	5.4
Standard random effects^2^	50	30.1
Marginal analysis	3	1.8
Bayesian methods	6	3.6
Multiple analysis methods	33	19.9
Other	1	0.6
Not stated	5	3.0
Meta-regression only	1	0.6
Heterogeneity test only	1	0.6
Unclear	3	1.8

^1^Out of 166 included reviews.

^2^See text for definition.

As seen in Table 5, fixed and random effect(s) models were used with roughly equal frequency. The term ‘standard fixed effect’ was used when the authors chose an accepted fixed effect model, such as the Mantel–Haenszel model [176], the inverse variance model or the Peto model [177]. Also, if the authors used any referenced fixed effect model this was recorded as ‘standard fixed effect’. In several cases the authors had used a method of combining data that would be considered as a ‘fixed effect’ model, but appeared to have either used a mean or weighted mean, or a logistic regression method, or had devised their own method for combining data, for example based on sample size of the studies. Such methods were considered non-standard and were recorded as ‘other fixed effect’. Interestingly, all the random effects models were referenced standard models (referred to as standard random effects in Table 5, such as the DerSimonian & Laird model [178]).

A record of ‘Multiple analysis methods’ was only selected when there was more than one method used on an equal basis. If there was an obvious primary method with additional supplementary methods the primary method was chosen.

The one meta-analysis reference referred to as ‘Other’ used a novel approach developed by the authors of ‘summary ranking’ involving assigning a score to the rank order of toxicity in individual primary studies, and then combining the scores to provide an overall rank order for toxicity. One study included a meta-regression as the only quantitative analysis, whilst another study performed a test for heterogeneity but did not produce any pooled estimates. In only three cases was there insufficient detail regarding the methodology to allow the type of analysis to be determined. In five of the reviewed studies the meta-analysis method was not stated.

A Bayesian approach was used by six included reviews, but in some cases the Bayesian model used in the meta-analysis was not fully described. Semi-Bayesian methods, incorporating a Bayesian use of a prior distribution on the rate of the adverse event alongside standard frequentist methods, were also used in one included review. This meta-analysis also experimented with the use of different prior distributions for the parameters. However, none of these studies presented graphical representations of the probability densities for the parameters.

Not taking into account the specific method of the meta-analysis, 74/166 (44.6%) used a fixed effect model. This included one review that stated that a random effects model was used, but in the absence of heterogeneity the presented results were fixed effect. (The novel method using rank summaries was also a fixed effect approach.) Three included reviews where the methodology was not described in sufficient detail to be classed as a standard or other fixed effect model, were, however, able to be classed as fixed effect in their approach.

A random effects model was used by 53/166 (31.9%) included reviews, including four of the reviews that used Bayesian methods. Hence, it is apparent that fixed and random effect(s) models were used with roughly equal frequency.

Both fixed and random effect(s) models were used in 28/166 (16.9%) included reviews, including one that used Bayesian methods. In the other cases it was not applicable (5/166; 3.0%), or not stated (5/166; 3.0%). It was unclear whether fixed or random effect(s) had been used in one included review only.

The reasons why the authors chose a particular model were recorded in 69/166 (41.6%) included reviews. Reasons based upon heterogeneity (or between-study variation) were the most commonly cited (46/69; 66.7%). Increased conservatism (of a random effects model) was also frequently mentioned; 7/69 (10.1%) reviews alluded to this.

Other reasons cited in support for a particular meta-analysis method included differences in primary study types, and so that larger studies would contribute more to the meta-analysis. Arriving at similar results from both fixed and random effect(s) models was also used to justify the chosen approach. Only one included review offered multiple explanations, while nine offered an explanation not mentioned above.

#### Type of primary study and approaches to inclusion

The types of primary study encountered by included reviews are set out in Table 6. The most frequent study type was some form of trial, the sole study type for 46.4% (77/166) of included reviews. Trials were clearly defined as being randomised and/or controlled in some included reviews. The 26 reviews (26/166, 15.7%) that included both trials and observational studies demonstrated a wide variety of approaches taken to this situation, often reflecting the number of each different type of study. Some included reviews made no attempt to differentiate by study design. In some included reviews there was only one trial, all other studies were observational, and in one of these reviews, the trial was excluded, although its inclusion did not alter the results. In another instance with only one trial, it was excluded from all meta-analyses, only the observational studies (of different designs) being included. In one included review the sole trial was excluded due to no events being observed in one group of the trial; similarly, in another review with only one trial, this study was excluded due to the small number of outcome events.

**Table 6:** Primary study types incorporated within included reviews

**Study types**	**No. included reviews**	**% included reviews**^**1**^
Randomised trials	70	42.2
Other trials^2^	7	4.2
Observational studies	56	33.7
Mixed (trials and		
observational studies)	26	15.7
Not stated	7	4.2

^1^Out of 166 included reviews.

^2^May include randomised trials but not specifically stated as such.

The most common approach to mixed study types was to perform a sensitivity analysis by analysing all primary studies together and then dividing the studies by some element of study design. For example, one included review combined all studies together and then case–control studies were analysed separately, and cohort studies were combined with trials. Some variation on this theme was followed by several other included reviews. Another approach was to avoid combination of estimates across study designs altogether, by combining results from studies with similar designs.

#### Graphical representations of data

Graphical representations of data were used in the majority of included reviews. Forest plots were the only graph used in 53.0% (88/166) of reviews, while meta-regression plots were the only plot in 1.8% (3/166). Both forest plots and meta-regression plots appeared in 1.8% (3/166) of included reviews. Other plots were used in 18.1% (30/166) of included reviews, usually a plot of the individual studies but lacking a pooled estimate. Only 25.3% (42/166) of included reviews produced no graphical representations of their results.

### Publication Bias

Publication bias was considered in 89/166 (53.6%) of included reviews. This issue may not have been specifically described in terms of publication bias. For example, some included reviews performed searches for unpublished primary studies, indicating that publication bias was within the awareness of the authors when performing a meta-analysis even if it was not taken any further than searching for such primary studies.

Publication bias was discussed but not formally evaluated in 44/166 (26.5%) included reviews. A quantitative analysis was performed by 31/166 (18.7%) included reviews. A sensitivity analysis by publication status was the preferred method of investigating publication bias for one review.

Of 31 included reviews with some form of quantitative analysis, 12 (38.7%) used a test with a *p*-value. The other 19/31 (61.3%) used an alternative method not resulting in a *p*-value. The most commonly used tests were Egger’s test [179] and Begg’s test [180]. Kendall’s tau test was mentioned by three included reviews.

The trim and fill method [181] was used to adjust for publication or selection bias in two included reviews. These were the only reviews that attempted to adjust for these types of bias. Graphical methods (funnel plots) were used to investigate for publication bias (or selection bias) in 29/166 reviews (17.5%).

The vast majority of included reviews used only published primary studies (129/166; 77.7%). Published primary studies with unpublished data (obtained through contact with the authors), were used in 20/166 (12.0%) included reviews. Both published and unpublished primary studies were used in 14/166 (8.4%) reviews. In the other reviews the study source(s) was either unclear or not stated. In several reviews where published primary studies only were included, it was made clear that unpublished data had been sought.

### Heterogeneity

Heterogeneity was considered in some manner by 138/166 included reviews (83.1%), whether by a quantitative or qualitative assessment, or by a subgroup analysis or meta-regression. Meta-regression was included in nine reviews that had no other assessment of heterogeneity, whilst one review discussed issues regarding combination of primary studies with different criteria, but did not do a formal qualitative or quantitative analysis of heterogeneity. Of the remaining 128 included reviews, a quantitative assessment was performed in 124 (124/166; 74.7%). A qualitative assessment of heterogeneity (for example, inspection of forest plots or noting heterogeneous results) was made in 10/166 (6.0%) included reviews. Six included reviews incorporated both quantitative and qualitative aspects of heterogeneity assessment (6/166; 3.6%).

Considering quantitative analysis methods, 121/166 (72.9%) included reviews employed some form of statistical test for heterogeneity, although with variation in the chosen critical *p*-value for significance. The chosen significance value was 0.05 for 28 reviews (23.1% of the 121 studies that performed a test), while 23/121 chose a more liberal *p*-value of 0.1 (19.0%). Only one included review chose 0.2 as the cut-off *p*-value. In many reviews the actual *p*-value was quoted without reference to a particular threshold (51/121 reviews (42.1%) did this). In the other reviews no *p*-value or significance level was stated.

An estimate for heterogeneity was presented by 16/121 included reviews with a quantitative analysis (13.2%). The most frequently-used estimate measure was the ^*I*2^statistic [182,183], which was used in 13 reviews. Alternative estimate measures included the between-studies variance. One review used another estimate measure, the R(I) statistic [184]. Only one included review used multiple estimate measures; the estimates used included ^*I*2^and the Q statistic [185].

Two ways to investigate the causes of heterogeneity are subgroup analysis and meta-regression. Subgroup analysis was performed in 27/128 (21.1%) reviews that included an analysis of heterogeneity, and in two reviews that did not formally assess heterogeneity. Meta-regression was used in 27 included reviews in total (27/166; 16.3%). In nine of these reviews, no formal assessment of heterogeneity had been performed. The covariates used in the meta-regression analyses were often very specific to the nature of the intervention or outcome being considered. A qualitative investigation of sources of heterogeneity was carried out in 17/138 reviews that considered heterogeneity in some way (12.3%).

### Individual patient data

Very little use was made of IPD in the reviewed meta-analyses. Only two included reviews of the total 166 (1.2%) included IPD. Of these two, all primary studies included had IPD available (so there was no requirement to combine IPD and summary data). Both reviews used a one-stage method for the meta-analysis. In one review the meta-analysis was stratified by trial and other factors including centre within study for multicentre studies and age divisions. In the other review it was not stated whether the meta-analysis was stratified or not.

### Sparse data

The issue of sparse data, whereby statistical methods were required to allow the inclusion of primary studies where the outcome was a count of zero, or a percent of zero, occurred in 65/166 included reviews (39.2%), one of which was a meta-analysis of single-arm studies only. Specific statistical methods may be required to allow incorporation of such a primary study into an overall pooled estimate or for calculation of confidence intervals. For 62 of the 65 reviews in which sparse data occurred, the outcome was on a comparative scale (an odds ratio, relative risk, or risk difference); this methodological area is the focus of this review. The primary issues are (i) outcome measures used by meta-analyses with zero events; (ii) use of continuity corrections; (iii) methods for inclusion of primary studies with zero events that do not involve continuity corrections; and (iv) incorporation of primary studies with zero events in both arms.

Of the 64 included reviews that considered the issue of sparse data in two-arm studies, 41 (64.1%) had datasets involving double-zero (zero events in both arms) primary studies. In the remaining 23 reviews, only single-zero (zero events in only one arm of a two-arm study) primary studies were present, or it was either unclear or not directly stated whether any double-zero studies were included within the dataset.

Of these 64 reviews, 30 (46.9%) presented their outcome as an odds ratio, 24 (37.5%) as a relative risk, and two as a risk difference. In six included reviews, there was more than one outcome with roughly equal importance in the meta-analyses. In two reviews, the outcome, where sparse data were incorporated, was an incidence rate.

Continuity corrections were used to circumvent problems with zero counts (in one or both arms of a study) that result in difficulties with estimating ratio-based outcome measures such as the odds ratio. Continuity corrections are also required to calculate the variance (and hence confidence intervals) for a risk difference. However, it was very difficult to determine an accurate picture of how continuity corrections were used.

Some included reviews clearly stated that continuity corrections had been used (15/64, 23.4%). In 17/64 (26.6%) included reviews, continuity corrections were not used. In 32 cases it was not clearly stated whether continuity corrections had been used or not.

The most popular primary continuity correction was 0.5, used in 14 of the 15 included reviews that stated their continuity correction. Only one meta-analysis reference used an alternative continuity correction with 0.25 being the chosen value. Only one review performed a sensitivity analysis across different continuity corrections, using 0.5, 0.1 and 0.01, and reported that the continuity correction did not alter the results. Three included reviews provided a reason for their choice of continuity correction, and the only reason cited was to minimise bias.

Several methods for incorporating single- and double-zero studies into a meta-analysis that do not involve continuity corrections were encountered in the included reviews. The most frequently used was the Peto method, employed by 12 studies. The use of a difference metric rather than a ratio as the outcome measure was used to circumvent problems with zeroes in seven reviews, although calculation of confidence intervals with such methods would be problematic. Seven included reviews resorted to the use of marginal analysis. Bayesian methods were used to tackle sparsity of events in only two included reviews.

Double-zero studies were included in analyses in 17 of the 41 reviews where double-zero primary studies were clearly present within the dataset. In two further reviews, double-zero studies were included in a sensitivity analysis. Double-zero studies were clearly excluded from analyses in 18 reviews where they were present in the set of primary studies. Such an exclusion was either a deliberate decision by the authors, shown by excluding the primary study in forest plots of the meta-analysis, or was done by default, the primary study being shown on a forest plot, but being given a weighting of zero. The four remaining included reviews were unclear as to whether or not these double-zero primary studies were included.

Of the 19 included reviews where double-zero primary studies were included in some way, seven made explicit use of continuity corrections. In other included reviews it was not clearly stated whether continuity corrections had been used. Other options, such as using a marginal analysis, were employed in some reviews, whilst in other reviews the methodologies used were unclear.

## Discussion

This paper has reviewed an extensive sample of published meta-analyses where the primary outcome was an adverse or unintended event. Reviews published in languages other than English were excluded, hence there is a risk that methodologies used in reviews published in other languages may differ from those discussed here. There was considerable variation in the methodology employed across the sample, including designs of the primary studies incorporated in the meta-analyses, use of fixed or random effect(s) measures, how to deal with zero events in study arms, and how to assess study quality and publication bias. In many cases, the statistical aspects were not clearly reported, with insufficient detail to discern the methods used. Often, little justification was given for the approaches to meta-analysis used. This is perhaps, in part, due to the lack of specific guidelines available for meta-analysis of sparse/adverse events. Not only would such guidelines improve the standard of reporting of adverse event meta-analyses, they could also circumvent disagreements in the literature due to the use of alternative methods producing different conclusions, as was the case for the recent high-profile concerns of elevated cardiovascular risk in those taking rosiglitazone [186-190].

Based on this review, one of the major areas of confusion appears to be with regard to the use of continuity corrections for dealing with sparse data for comparative outcomes. Indeed the term continuity correction is possibly misleading, as these are in effect nothing more than arbitrary factors added to a cell count of zero. Where a study has zero events in both groups, we believe it contains no information regarding the magnitude of the odds ratio or relative risk, but adding a continuity correction to both sides (incorrectly) keeps it in the analysis for methods which require it. Although not required for the risk difference scale, and such a study does contain information, a correction factor is required for the estimation of associated variance which causes further confusion. This latter point also raises the unanswered question of whether the presence of double-zero studies should influence the outcome metric. Bayesian methods present an alternative way to deal with datasets including sparse data, and Bayesian analyses are now easily implemented using appropriate software. However, Bayesian methods were used infrequently within the included reviews; with the development of guidelines to support Bayesian analyses, it may be that such methods would become more popular.

Many reviews in this sample included observational studies, in some cases this was the only type of primary study included, whilst in other reviews both observational studies and trials were included. Observational studies may offer advantages over trials, such as a longer period of follow-up. Inclusion of observational studies also increases the number of studies and individuals within a meta-analysis where both trials and observational studies are available, thus increasing power. However, this advantage may be counterbalanced by concerns regarding bias in observational studies. The reviews included here showed a variety of approaches to mixed study design; this is an area where guidelines would assist in combining all available data whilst addressing issues of different study design.

As mentioned in the introduction, a previous systematic review of reviews and meta-analyses of primary studies of adverse effects of a drug intervention has been conducted [4], including a total of 43 references, all published in 2006. Of these, 15% assessed quality of primary studies, compared to 42.2% of references in our review, and only 24 performed a meta-analysis. As seen in our review, there was some poor reporting of the methods used for pooling data, but 83% did report the method used for pooling data and exploring heterogeneity. With regard to funding source, 23% (of the 43 reviews) had pharmaceutical funding, compared to the 16.3% of references in our review that had commercial funding. Hence, there is potentially some suggestion of systematic differences between reviews concerning only drugs and those including other interventions, and between reviews that contain a meta-analysis and those that do not (although such observations could be confounded by the wider time range we considered).

### Development of guidelines

Within the field of meta-analysis for adverse events, the concept of definitive guidelines is possibly too prescriptive; the diverse nature of medical interventions, the potential adverse outcomes and the ways they may be measured, and the formats in which data may be available preclude the use of standardised methods. However, general guidelines for approaching specific methodological issues (applying to other outcomes beyond adverse events) may be more feasible and useful. We hope this review sets the context in which future research and guidelines into the conduct of adverse event meta-analyses can be placed.

There is perhaps a need for more research before informed guidelines could be drafted. Many of the unique issues relating to adverse event meta-analyses are due to the typically sparse event data available for such analyses. Such sparse data presents unique challenges, as highlighted by this review. As well as specific challenges, such as dealing with zero events in arms of studies, there are also potential concerns relating to the use of broadly accepted meta-analysis methods in a sparse data context due to the potentially very low power such methods may have. For example, simulation has shown that tests for heterogeneity have very low power in sparse data situations [191,192]. Similar issues are likely to exist for the use of meta-regression, tests for publication bias [193] and other methodologies. Rather than trying to use cutting-edge advanced meta-analysis methodology, in a sparse data context, it may often be wiser to restrict focus to simpler methods (e.g. fixed effect models) and be realistic about the potentially limited conclusions that can be drawn from the data. Since the primary aim of many adverse effect meta-analyses is to establish the existence of an elevated risk of an event due to a particular intervention, use of simple methods is, perhaps, consistent with this aim. Information on the performance of different meta-analytic estimators for sparse data and the use and avoidance of correction factors has been considered at length elsewhere [191,192] and this is one domain in which knowledge on which methods to use and which to avoid is available.

Hierarchical models to address issues related to combination of different study types have been developed [194], and more recent developments on the use of methods to adjust studies in meta-analysis to account for bias appear to be promising [195,196]. However, further exploration into the use of such methods in an adverse events context is warranted.

Development of Bayesian methods is clearly an area where further research would be both timely and beneficial, especially in the light of many of the difficulties surrounding meta-analysis of adverse events data, which Bayesian methods may be able to address, such as inclusion of primary studies with sparse events without the need for continuity corrections using Markov Chain Monte Carlo (MCMC) methods [197-200]. However, difficulties exist in ensuring that all prior distributions are *plausibly vague*, when not based on external information – a challenge given how little data is sometimes available [201,202].

Specifically to adverse events, an area where further research would be valuable is the consideration of drug class effects, allowing information on multiple drugs of the same class to be combined while acknowledging the potential differences in effects across drugs. This would be an area where hierarchical (and potentially Bayesian) models would be particularly useful [203]. Another clinical aspect related to adverse events issues is that there may be several indications for a certain intervention; patients with different conditions may be at varying risk of adverse events, despite receiving the same intervention. Whilst it is desirable to combine all available data to increase power, any meta-analysis should be able to adjust for the differences in indication for the intervention; as this scenario is similar to combination of data regarding individual drugs of the same class, hierarchical modelling may be a means to achieve this.

## Conclusion

Conducting meta-analyses where the outcome is an unintended or adverse event presents a range of potential difficulties, and requires careful consideration of the statistical issues, as well as an awareness of the clinical context. This review has demonstrated that a diversity of approaches have been employed when conducting such meta-analyses. Hence, standardised guidelines may be beneficial in this area, even if, due to the range of clinical situations and availability and format of data, they are necessarily of a general nature. This is especially true since a meta-analysis may present the only feasible method to estimate potential risks, due to the often infrequent occurrence of adverse events within an individual trial or observational study.

## Competing interests

The authors declare that they have no competing interests.

## Authors’ contributions

FCW selected and reviewed the included references, with guidance from KRA and AJS. SG developed the online search strategies and performed all literature searches (online and by hand). FCW drafted the paper, which was revised by all co-authors. All authors read and approved the final version of the manuscript for publication.

## Pre-publication history

The pre-publication history for this paper can be accessed here:

http://www.biomedcentral.com/1471-2288/12/64/prepub
